# Evaluation of QT Dispersion in Children with Breath Holding Spells

**Published:** 2016

**Authors:** Amir Hosein MOVAHEDIAN, Marzieh HEIDARZADEH ARANI, Davood MOTAHARIZAD, Gholam Abbas MOUSAVI, Ziba MOSAYEBI

**Affiliations:** 1 Department of Pediatric Cardiology, Tehran University of Medical Sciences, Tehran, Iran; 2 Department of Asthma, Allergy & Immunology, Kashan University of Medical Sciences, Kashan, Iran; 3 Pediatrician, Kashan University of Medical Sciences, Kashan, Iran; 4 Statistics, Kashan University of Medical Sciences, Kashan, Iran; 5 Department of Neonatology, Tehran University of Medical Sciences, Tehran, Iran

**Keywords:** Breath holding spell, Dispersion, QT interval, QT dispersion, QTc dispersion

## Abstract

**Objective:**

Breath holding spells (BHS) are common involuntary reflexes in infancy and early childhood. Differential diagnosis should embrace Long QT Syndrome (LQTS) and paroxysmal abnormalities of rhythm. The aim of this study was to compare QT dispersion (QTd) in children with breath holding spells and normal controls.

**Materials & Methods:**

QT dispersion and Corrected QT(QTc) dispersion were measured in 12 lead surface electrocardiograms in 56 patients with BHS and compared with healthy children of the same age referred to the clinic for regular checkup visits.

**Results:**

The most common type of BHS was cyanotic (83.9%). Seven patients (12.5%) had pallid and two patients (3.5%) had mixed spells. There was a history of breath holding spells in 33.9% of the children. QT dispersion was 61.6± 22.5 and 47.1±18.8 ms in patient and control groups, respectively. QTc dispersion (QTcd) was 104 ± 29.6 and 71.9 ±18.2 ms, respectively. There was a significant difference between patient and control groups in terms of QTd and QTcd (P<0.001).

**Conclusion:**

QTd and QTcd were increased in children with BHS. Therefore, the evaluation of EKG for early diagnosis of rhythm abnormalities seems reasonable in these children.

## Introduction

Breath holding spell is a sudden, reflexive and non-epileptic phenomenon that is frequent in infancy. Patients with this symptom usually refer to pediatric neurology and cardiology clinics for evaluation of seizure and heart disease (1-3). The spells usually start at early 6-12 months of life and resolve approximately at age 4-5 yr, but rare cases have been reported to persist until the age of 7 yr in about 4-5% of children older than 8 yr, that might be afflicted with BHS. Hereditary background Autosomal Dominant (AD) has been a cause attributed to this disease (1, 3, 4). Although the etiology is not known, autonomic dysfunction and increase vagal tonus leading to cardiac arrest and cerebral anoxia are considered to play a role. This problem can gradually resolve with autonomic system maturation (1, 5, 6). Relation between iron deficiency anemia and BHS has been reported, but how iron deficiency leads to BHS is a matter of challenge (2). Diagnose usually starts with the observation of typical spells. The spells always occur in waking hours followed by crying and silent expirations. Skin color changing to pallid or cyanotic leading to loss of consciousness and decrease of tone, have been reported in conjunction with these symptoms. The symptoms resolve spontaneously (4). Based on changes in skin color during the spell, BHS can be divided into two groups of cyanotic and pallid. However, sometimes children may suffer from a mixed type (1, 4). Cyanotic episodes are more common than pallid episodes. Excessive parasympathic sensitivity can cause bradycardia, decrease of cardiac output, and blood pressure that finally results in pale look. The treatment of BHS focuses on supporting and ensuring parents (1, 3, 4). Spells were formerly believed to resolve with the passage of time and the evolution of autonomic system; however, further studies revealed different prognostic results for these children including the higher probability of faint and sinus arrhythmia in future than in their healthy peers (6-9). QT dispersion is a useful way of non-invasive evaluation for repolarization of ventricular myocardium through superficial ECG, which also shows the risk of serious ventricular arrhythmia and coronary artery disease. Increase of QT dispersion, which increases the risk of dysrhythmia and sudden death, can be seen in different diseases such as cardiomyopathy, mitral valve prolapse, ischemic heart disease and renal failure (10, 11). In Turkey, QT significantly increased in patients with BHS (1), but in another study, no difference was observed in QT between healthy children and children with BHS (12). Because of the mixed results of the studies on BHS and the infrequent evaluation of prolonged QT in previous research, the discovery of prolonged QT syndrome and its possible health risks for children is necessary. In this study, we evaluated QT dispersion for abnormal ventricular repolarization and rhythm in children with BHS and compared QTd, QTcd, and QTc values in patients with cyanotic, pallid and mixed spells.

## Materials & Methods

This case-control study includes 56 children from 3 mo-old to 6 yr-old age diagnosed with BHS at Shahid Beheshi Pediatric Clinic, Tehran, Iran from June 2010 to September 2011. Enrlloed children under were diagnosed by pediatric cardiologists and neurologists with cyanotic or pallid spells. All other etiologies had been excluded and BHS was highly probable. EKG and echocardiography were taken from all children. Patients suffering from an organic disease or a disease affecting EKG and those consuming QT-affecting drugs (e.g. erythromaycin, clarythromycin, and furosemide) were excluded from the study. The control group included 56 healthy male and female children aged three months to six years of no history of using QT-affecting drugs. They referred to the clinic for regular checkups. The control group and the case group were matched. EKG with 12 leads was taken in this group. Parents of the children participated in the study were taken informed consent. The study was approved by the Ethics Committee of Kashan University of Medical Sciences, Kashan, Iran. The 12 lead ECG of the entire case and control group were obtained using a Bionet cardiocare electrocardiography machine; RR and QT intervals, QTd, QTc and QTcd were measured by a pediatric cardiologist. To evaluate heart disease, echocardiography was taken only from children with BHS using ATL Philips/HDI-3500cv echocardiography machine. QT interval was measured as the interval between QRS complex and the end of the “T” wave. The point where the wave returns to the isoelectric line was considered as the end of the “T” wave. RR and QT intervals were measured precisely using a special ruler. QTc was calculated through the Bazet formula ($\mathrm{QTC}=\frac{\mathrm{QT}}{\sqrt[{2}]{R-R}}$) QTd was then calculated as the difference between the longest and the shortest QT intervals. QTcd was measured in the same way. All the measurements were done by one person in 12 leads. QTcd, QTd, QTc and QT interval values were measured and recorded for control group and patients with cyanotic, mixed and pallid spells. As for statistical analysis, the variables and EKG information for patients and the control group were analyzed using statistical tests such as Chi-Square, Mann-Whitney, Levine’s, T, Kolmogorov-Smirnov and Kruskal-Wallis.

## Results

Out of 56 case group, 26 (45.5%) were male with an average age of 20.03 ± 13.67 mo. The mean age of breath holding spell in the case group was 8.4 ± 7.03 mo. Forty-seven patients had cyanotic, seven patients pallid and two patients mixed spells. 33.9% of the children had the history of breath-holding spells in their family. The highest mean QT values were 343.3 in the case group and 300.1 in the control group. The lowest mean QT values in the case group and in the control group were 281.4 and 252.9, respectively. The highest values for QTc in the case group and in the control group were 498.9 and 444.8 and the lowest values were 394.6 and 372.9, respectively. All these differences between the groups were statistically significant (P<0.001). Mean QTd among the children in the case group was 54.4 ± 21.9 ms and mean QTcd was 88.1 ±29.3 ms. These values were lower in the control group (Table 1 and 2). QTd and QTcd mean differences between the two groups were statistically significant as well (P-value<0.001). No statistically significant correlation was found between sex, spell intensity, spell history, consciousness, and tone on the one hand and QTd and QTcd on the other hand (P-value>0.05). Echocardiography in the case group (afflicted with BHS) was normal indicating no heart disease.

**Table 1:** Descriptive Statistics for Age, Onset of BHS, QTd, and QTcd in Case and Control Groups

**QTcd (ms)**	**QTd (ms)**	**Onset of BHS (mo)**	**Age (mo)**	**VariablesMD±SD**
104.2±29.6	61.6±22.5	8.4±7.03	20.03±13.6	**Case**
71.9±18.2	47.1±18.8	-	20.01±13.6	**Control**

**Table 2:** Status of QT, QTc,QTd, QTcd in Children under the study

**Control group**	**Case group**	**Variables**
300.1±29.6	343.3±36.5	**The most frequent amount of QT(ms)**
252.9±24.4	281.4±42.3	**The least frequent amount of QT (ms)**
444.8±22.9	498.9±25.2	**The most frequent amount of QTc(ms)**
372.9±21.8	394.6±29.9	**The least frequent amount of QTc(ms)**
47.1±18.8	61.6±22.5	**QTd (ms)**
71.9±18.2	104.2±29.6	**QTcd(ms)**

Statistical comparison (*P *value) <0.001

**Table 3:** QTd and QTcd Values of the Case Group Based on the Type of the Spell and Its Frequency

**Variables**		**QTd (ms)**	**QTcd(ms)**
**Kind of spells**	Cyanotic	59.8±19.7	97.3 ± 26.1
	Pallid	80 ± 32.6	139.3± 21.8
	Mixed	40 ± 0	143.1 ± 0
	Statistical comparison	0.03	<0.001
**Frequency** **Of spells**	More than one spell a day	80± 0	141.5 ± 21.3
	Daily	72.7 ± 30	118.1 ± 26.7
	Weekly	55.6 ±19.6	88.9 ± 30.1
	Monthly	55.8 ± 19.2	102.5± 22.9
	First spell	80 ± 0	121.1 ± 0
	Statistical comparison	NS	NS

## Discussion

The prevalence for different types of spells in this study was 83.9% for cyanotic spells, 12.5% for pallid spells, and 3.6% for mixed spells. In Akalin et al., study, the prevalence was 72.09% for cyanotic spells, 18.6% for pallid spells and 9.3% for mixed spells (1). 33.9% of the children in the case group had positive family history of BHS. In previous studies, breath holding spell history among family members of the participants afflicted with BHS was reported as 23% (6, 13, 14). The means for QT and QTc variables for children with BHS were significantly higher than those of healthy children were. This was in contrast with findings reported earlier, where no significant difference was observed between QT and QTc intervals in patients with BHS and healthy children (1, 12). In Kolkiran et al., study, iron deficiency was also considered as a contributing factor. DiMario reported higher QTc in children with BHS than healthy children (15).Similarly, in the present study mean QTd was 61.6± 22.5 for the case group; whereas, for the control group, it was 47.1± 18.8. Mean QTcd was 104.2± 29.6 for the case group and 71.9 ± 18.2 for in control group. Based on these findings, QTd and QTcd values significantly increased in children with BHS. In Akalin et al., study, average QTd was 59.5± 35.9 in the case group and 44.8± 11.9 in the control group (1). Average QTcd in that study was 102.1± 41.9 for the case group and 79.6± 24.6 for the control group. The differences that were reported to be significant are similar to our findings and support our results. Our results contradict with those reported in Kolkiran et al., study where no differences in QTd and QTcd values were found among children with BHS and healthy children (12). In our study, QT, QTc, QTd and QTcd values in children with the pallid type of BHS were higher than those of healthy children were. In previous study, no differences were identified between cyanotic and pallid types of BHS, even though the rate of sinus arrhythmia and asystole during BHS was higher in the pallid type (12). In another study of BHS, DiMario reported higher QTc in children with the pallid type of BHS than in children with the cyanotic type of BHS (15). He also reported higher probability of sinus arrhythmia in children with the pallid type of BHS than in the control group or in cyanotic BHS type group (9). Similarly, Alkalin et al., found higher QTd and QTcd rates in children with pallid and mixed types of BHS. Our findings support those reported in their studies (1). Based on the convergent findings of these studies that point to relationships between prolonged QT syndrome (LQTS) and BHS (7, 9, 16), it is suggested that prolonged QT syndrome and sudden abnormalities of rhythm should also be considered in the differential diagnosis of BHS. In America, electrocardiography is essential in discovering prolonged QT syndrome cases (7). In this study, significant correlations were found between the frequency of BHS on the one hand and QTd and QTcd values on the other hand (P-value<0.05). Accordingly, these values should be considered in children with high frequency spells.

**In conclusion**, QT, QTc, QTd and QTcd variables were all higher in the children with BHS than in healthy children. There was also a significant relationship between pallid type of the spells and frequencies of more than one time a day and QT and QTC values. Because of the possibility of the incidence of prolonged QT syndrome in children with BHS and consequently higher probability of sudden death, exact and in time evaluation of ECG and especially of QT interval in children with BHS seems vital. To prevent probable sudden deaths, these should be given due attention especially in the case of the pallid type of BHS and with a frequency of more than one spell a day.

## Authors’ contribution:

Amir Hosein Movahedian: Study concept and design 

Marzieh Heidarzadeh Arani: Study concept Davood Motaharizad: Acquisition, analysis and interpretation of data 

Gholam Abbas Mousavi : Statistical analysis 

Ziba Mosayebi: Drafting and revision of the manuscript All authors agreed to be accountable for all aspects of the work in ensuring that questions related to the accuracy or integrity of any part of the work are appropriately investigated and resolved.
